# Unlocking the biodegradative potential of native white-rot fungi: a comparative study of fiberbank organic pollutant mycoremediation

**DOI:** 10.1080/21655979.2024.2396642

**Published:** 2024-09-02

**Authors:** Burcu Hacıoğlu, Gabriel Dupaul, Gabriela Paladino, Mattias Edman, Erik Hedenström

**Affiliations:** Department of Natural Sciences, Design and Sustainable Development, Mid Sweden University, Sundsvall, Sweden

**Keywords:** Bioremediation, mycoremediation, fiberbank, white-rot fungi, diplomitoporus crustulinus, phlebia tremellosa, phlebiopsis gigantea, organic pollutants

## Abstract

Fiberbanks refer to a type of fibrous sediment originated by the forestry and wood pulping industry in Sweden. These anthropogenic sediments are significantly contaminated with potentially toxic elements, and a diverse array of organic pollutants. Additionally, these sediments are of environmental concern due to their potential role in greenhouse gas emissions. Given the environmental risks posed by these sediments, the development of effective remediation strategies is of critical importance. However, no specialized methods have been established yet for the cleanup of this specific type of contaminated sediments. To identify effective fungal species for the mycoremediation of the fiberbank substrate, we performed a detailed screening experiment. In this research, we primarily aimed at assessing both the growth capacity and the proficiency in degrading organic pollutants of 26 native white-rot fungi (WRF) species. These species were sourced from natural forest environments in northern Sweden. The experimental setup involved evaluating the WRF on plates containing fiberbank material with a central Hagem-agar disc to closely monitor the interaction of these species with fiberbank substrates. Among the fungi tested, *Laetiporus sulphureus* exhibited the highest growth area percentage at 72%, followed by *Hymenochaete tabacina* at 68% and *Diplomitoporus crustulinus* at 67%. For the removal of 2–3 ring polycyclic aromatic hydrocarbons (PAHs), *Phellinus punctatus* led with 68%, with *Cystostereum muraii* at 57% and *Diplomitoporus crustulinus* at 49%. Regarding the removal percentage of 4–6 ring PAHs, *Diplomitoporus crustulinus* showed the highest efficiency at 44%, followed by *Phlebia tremellosa* at 40% and *Phlebiopsis gigantea* at 28%.

## 1. Introduction

Fungi play a pivotal role in the biodegradation of organic pollutants, primarily due to their sophisticated enzyme systems. These organisms possess the capability to decompose a wide spectrum of organic pollutants owing to their extracellular enzymatic activities [1,2,3]. Specific enzymes, including laccases, manganese peroxidases, and lignin peroxidases, have been identified as particularly efficacious in breaking down organic pollutants [4–6]. Besides enzymatic degradation, the mycelial networks of fungi serve as biofilters, facilitating the accumulation and absorption of contaminants. Subsequently, these absorbed substances can undergo metabolism and further degradation through the action of intracellular enzymes [1].

Numerous factors influence the degradation rate of organic pollutants by fungi, including nutrient availability, co-substrates, pH, temperature, and competition from other microorganisms [7]. Despite their potential, challenges remain in maintaining fungal viability in contaminated environments and scaling from lab to field applications. However, mycoremediation, the use of fungi for bioremediation, shows significant promise [1,3,8]. Mycoremediation is an environmentally friendly technique offering a cost-effective and sustainable solution for cleaning up contaminated environments. Additionally, it is safe, versatile, and well-received by the public, with benefits for ecological restoration [9–11].

White rot fungi (WRF) are a distinct group of fungi recognized for their capacity to decompose lignin, effectively breaking down lignocellulosic constituents within plant materials. This capability is critical for nutrient recycling within forest ecosystems, although the exact contribution of WRF to carbon cycling within these ecosystems remains to be fully elucidated [1,12,13]. WRF produce specific enzymes, namely lignin peroxidase, manganese peroxidase, and laccase, which are instrumental in lignin degradation. These enzymes are particularly notable for their ability to degrade a wide array of structurally diverse environmental organic pollutants, underscoring the unique role of WRF in this context [4,14]. The capability to synthesize these enzymes positions WRF as key organisms in the bioremediation of contaminated ecosystems, effectively breaking down pollutants such as polycyclic aromatic hydrocarbons (PAHs) [15], aliphatic hydrocarbons [16], chlorinated compounds [17], dioxins, various pesticides [18], and a range of xenobiotic molecules [2].

The extracellular enzymatic system of WRF can oxidize a broad spectrum of PAHs through a process that initiates with the oxidation of a PAH molecule, leading to the formation of less complex intermediates. These intermediates can be further broken down more readily. Given the nonspecific nature of this enzymatic activity, WRF can target a wide range of PAHs, enhancing their utility in environmental remediation efforts [4,19].

WRF can break down aliphatic hydrocarbons, which are carbon and hydrogen compounds found in petroleum. These hydrocarbons degrade through pathways similar to those for PAHs, involving enzymes like peroxygenases, cytochrome P450, and laccases. These enzymes initiate oxidation, fragmenting the hydrocarbons for further metabolism by fungi or other microbes [20–23].

In Sweden, before more stringent environmental regulations were endorsed in 1969, a significant amount of wood-derived fibers, along with chemicals used in the pulping process, were often discharged into nearby water bodies with wastewater from the pulp and paper industries. ‘Fiberbanks,’ as commonly referenced in Scandinavian literature, are thick deposits of polluted wood fibers and scraps that originate from these wastewater discharges. These deposits are typically found in aquatic environments near former or current pulp and paper mills. Fiber-rich sediments are natural mineralogenic sediments enriched with polluted fibers that can be found around fiberbanks [24–26]. Fiberbanks and fiber-rich sediments cover an approximate surface area of 2,500,000 m^2^ and 26,500,000 m^2^, respectively, in the water bodies in Sweden, including the Baltic Sea [27,28]. However, this issue is not limited to Sweden, as countries like Finland, Norway, and Canada also have water bodies polluted with these types of materials [29,30].

These anthropogenic sediments, known as fiberbanks, can contain a variety of organic and inorganic pollutants, such as residual lignin, wood-derived organic compounds, paper-making additives (fillers, coatings, sizing agents), and chemicals from pulp bleaching (including chlorine and chlorine-derived compounds, potentially forming dioxins). They also contain PAHs and heavy metals like mercury and cadmium used in different stages of paper production or present in the wood. The presence of aliphatic and aromatic hydrocarbons in fiberbanks is often due to fuel and oil leakages from pulping factory operations and nearby industries [27,28]. The environmental concern with fiberbanks lies in their potential to be long-term pollution sources due to the slow diffusion of contaminants into aquatic environments. Disturbance of these sediments, whether through natural events or human activities, can lead to the remobilization of particles and the release of pollutants into the water column, posing risks to aquatic life and potentially affecting water quality for human use [24,30–32].

Management and remediation of fiberbank sites involve assessing pollutant risks and deciding on appropriate actions, which may include monitoring, containment, in-situ treatment, or sediment removal for ex-situ treatment and disposal. The chosen strategy depends on the nature and level of contamination, proximity to human populations, and environmental protection priorities [33]. For ex-situ treatment, bioremediation methods are environmentally sound options for detoxifying and reusing or disposing of these sediments. Fiberbanks and fiber-rich sediments are unique due to their high organic matter content, mixed pollution, particle size, and heterogeneity. Given the lignocellulosic material in these sediments, exploring their mycoremediation using WRF is particularly interesting, despite the lack of published attempts using fungi for the bioremediation of these materials.

Our study aimed to achieve two goals: (i) to assess the biodegradative capacities of organic pollutants of a comprehensive selection of 26 native WRF species isolated from nature in the county of Västernorrland (Sweden) and (ii) to evaluate the potential utility of these species in the context of fiberbank material’s organic pollution remediation. In pursuit of these goals, we conducted a complete screening study to assess the biodegradative capacities of targeted organic pollutants of the isolated native WRF species, employing fiberbank substrate as the test material.

## 2. Materials and methods

All research reported in this paper has been conducted in an ethical and responsible manner and is in full compliance with all relevant codes of experimentation and legislation. The fungal samples used in this study were collected from forests in Sweden. According to the regulations and guidelines governing such activities, no special permissions were required for the collection of these fungi.

### 2.1. Fiberbanks collection and characterization

Fiberbanks material was collected from Sundsvall’s Bay, located in the central region of the Baltic Sea in Sweden. Over time, the bay has hosted numerous factories engaged in various industrial activities, resulting in significant emissions impacting the area. Among these, two prominent factories are believed to be the primary sources of fibrous sediment emissions: the Ortviken sulfate pulp factory and a sawmill located northwest of it. The Swedish Geological Survey [27] identified and monitored five distinct fiberbanks, each surrounded by sediments rich in fibers, in the vicinity.

**Figure 1.**
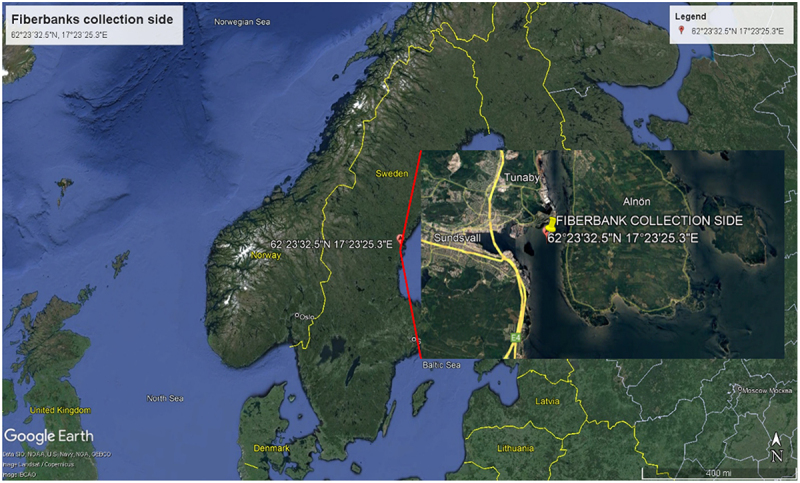
The designated site for fiberbanks sampling is located within Sundsvall Bay, strategically positioned along the coastline of the Bothnian Sea in Sweden. This specific area, employed for mycoremediation experimental studies, is distinctly identified by a red square on the geographical mapping. (source: Google earth, https://earth.google.com/web).

One specific fiberbank, situated toward the eastern end, spans an impressive 45,000 square meters and boasts a pure waste cellulose fiber layer approximately 6 meter thick. For this study, a bulk sample of around 500 liters was procured from this fiber deposit. The collection point, located at coordinates WGS84: 62°23´32.5” N, 17°23´25.3” E, was at a water depth of 18 meters, specifically at the top of the fiberbank (Figure 1). The collection was executed using a two-meter-long core tube sampler, facilitating gravity-driven dewatering. The extracted material was then subjected to a dewatering process within a cylindrical container equipped with a bed of gravel and geotextile, allowing for effective dewatering over 5 days.

After the dewatering process, the resultant fiberbank material, both fresh and sterilized through autoclaving, was utilized in the experimental procedures. Representative samples were obtained from the fresh dewatered fiberbank substrate. These samples were stored at a temperature of −20°C to ensure their preservation for later chemical analysis.

An accredited external laboratory facility (Eurofins Environment Testing Sweden AB, accreditation no. 1125 ISO/IEC 17025) performed a comprehensive environmental hazard screening, assessing a total of 161 parameters. Given the limited existing information on fibrous sediments, this comprehensive screening encompassed various aspects, including aliphatic hydrocarbons, PAHs, volatile organic compounds (VOCs), phenols, organochlorine pesticides, polychlorinated biphenyls (PCBs), selected herbicides, methylmercury, toxic metals, and metalloids.

The outcomes of these analyses are presented in the supplementary material (Table SM-1 for samples before autoclaving, and Table SM-2 for samples after autoclaving), providing valuable insights into the concentrations of target pollutants. There was a noticeable reduction in the concentrations of alkanes and aliphatics after autoclave treatment. This outcome was also observed in PAHs, affecting both low (LMW) and high molecular weights (HMW). Slight variations were detected in the levels of potentially toxic elements (PTEs).

### 2.2. Fungi collection and inoculation

#### 2.2.1. Fungi collection

Fungal strains used in the experiment were isolated from fruiting bodies of WRF collected on various natural wood substrates in the boreal forests of northern Sweden. In total, 26 species were included (Table 1). The species represent a variety of ecological functional traits, such as host tree specialization and preferred wood decay stage (primary-, secondary- and late colonizers).

**Table 1.** The included 26 species of WRF and adherent GenBank accession numbers when available.

Species	GenBank
*Bjerkandera adust*a	–
*Cystostereum muraii*	–
*Diplomitoporus crustulinus*	AF343320
*Diplomitoporus lindbladii*	AJ006682
*Ganoderma applanatum*	
*Hapalopilus aurantiacus*	AY986499
*Heterobasidion annosum sensu lat.*	X70027
*Hymenochaete tabacina*	–
*Ischnoderma benzoinum*	–
*Junghuhnia collabens*	–
*Junghuhnia luteoalba*	–
*Laetiporus sulphureus*	–
*Meruliopsis taxicola*	AY787673
*Phellinus chrysoloma*	–
*Phellinus ferrugineofuscus*	JQ518285
*Phellinus nigrolimitatus*	AY558632
*Phellinus populicola*	–
*Phellinus punctatus*	–
*Phellinus viticola*	–
*Phlebia tremellosa*	–
*Phlebiopsis gigantea*	DQ320133.
*Skeletocutis odora*	–
*Stereum rugosum*	–
*Stereum sanguinolentum*	–
*Trametes hirsuta*	AB158313
*Trametes ochracea*	–

The identities of nine of the isolated species were confirmed by DNA sequencing as part of another project. The identities of the rest of the species were confirmed by their morphological characteristics. We mainly looked at the color and texture of the aerial mycelium, but in some cases, we also took samples for microscopic identification [34]. Mycelial characteristics were distinct and evident, making the species confirmation straightforward.

#### 2.2.2. Fungi inoculation

Twenty-six collected fungal species were cultured in a Hagem agar medium consisting of 20 g agar, 5 g glucose, 5 g malt extract, 0.5 g NH₄NO₃, 0.5 g MgSO₄·7 H₂O, and 0.5 g KH₂PO₄ per liter. This pre-cultivation process lasted for two weeks at room temperature (~21°C) before the subsequent inoculation onto the fiberbank material. The experimental design aimed to observe fungal attraction to the fiberbanks substrate and assess their capacity to interact with and metabolize these substrates.

**Figure 2.**
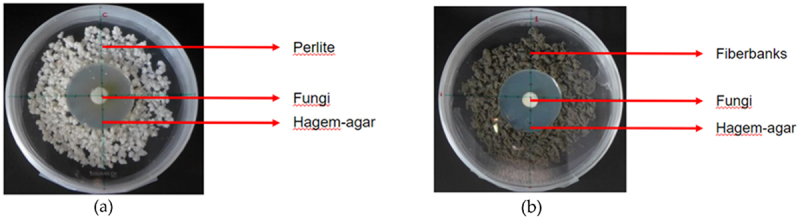
The experimental setup is illustrated through two distinct configurations as depicted in the accompanying images. Image (a) highlights the control samples, containing a layer of perlite with a central section of Hagem agar. Above this agar, centrally positioned, is a disc of fungal mycelium. In contrast, image (b) presents the treatments setup where perlite is replaced by fiberbanks material yet retains the same configuration of the centrally placed hagem-agar and the fungal mycelium disc positioned at the center and top of the agar. (photos: Burcu Hacıoğlu).

Working in a sterile laminar flow cabinet, a circular Hagem agar disc with a radius of 5 cm was prepared and positioned at the center of a 16 cm diameter Petri plate. The sterilized fiberbank material, previously autoclaved at 121°C for 20 minutes, was uniformly distributed around the Hagem agar disc, extending until it occupied an approximate radius of 15 cm. The fungal specimens, sourced from the Hagem agar plates, were collected using a sterile pipette with a radius of 5 mm. These specimens were then introduced at the midpoint of the Hagem agar (Figure 2b). Three replicates with the fiberbank substrate were made for each species.

For the control samples, perlite sterilization was conducted identically to the treatment of fiberbanks substrate, utilizing autoclaving at 121°C for 20 minutes. Under the same working conditions, control samples were prepared. Perlite, serving as the substitute for fiberbank material, was evenly dispersed around the Hagem agar to cover an approximate diameter of 15 cm, as depicted in Figure 2a. A single replicate was prepared for each species to serve as the control sample.

### 2.3. Fungi incubation and growth in fiberbanks material

After inoculation, the plates were sealed with parafilm and incubated at room temperature (~21°C) with regular daylight for two months. The progression of fungal growth was consistently monitored, with photographs taken weekly using a Leica D-LUX 6 camera. The extent of fungal mycelium expansion was quantified utilizing ImageJ (1.53g60) software [35,36]. To ensure consistency and eliminate environmental interference, these photographs were captured within a controlled setting of a Puluz Photo Light Box with medium flush light. In ImageJ, the area covered by the mycelium was determined through manual delineation of the images.

After a two-month growth period, the substrate from the fiberbanks was collected around the Hagem agar, without separating the mycelium. The harvested fiberbank substrate was initially subjected to freeze-drying for 48 hours, followed by homogenization using a Precellys Evolution homogenizer (15 mL tube, 7500 RPM, cycle 2 × 20 s, pause 15 s) with bulk beads (2.8 mm zirconium oxide beads). Subsequently, each sample of the fiberbank material was partitioned into three aliquots in preparation for the extraction of organic pollutants. These samples were preserved in cold storage at 4°C until the time of analysis.

### 2.4. Organic pollutants analysis

Organic pollutants were extracted from 2 g of freeze-dried specimens originating from the fiberbanks substrate, and perlite-encompassing samples prior to and after fungi growth. This extraction procedure was conducted utilizing accelerated solvent extraction (ASE 350, Dionex Corp., Sunnyvale, USA). Two extraction cycles were employed, each employing distinct solvent systems: dichloromethane:hexane (1:1 v/v) and hexane:acetone (1:1 v/v). The ASE process was conducted at a temperature of 100°C, with a static time of 10 minutes for the initial cycle and 5 minutes for the subsequent cycle. The extracted samples were concentrated and enriched with 10 µg of 2-fluorobiphenyl and 1-chlorooctadecane, acting as surrogates for PAHs and aliphatic hydrocarbons, respectively (according to USEPA Methods 8100 and 8081b). Subsequently, extract purification was executed utilizing a pre-conditioned 1 g silica gel cartridge (Strata® SI-1 Silica), employing a 12 ml solution of dichloromethane:hexane (1:1 v/v) for elution. For sulfur removal, activated copper beads were introduced to the concentrated extract and subjected to vortexing. The purified extract underwent further concentration, followed by solvent exchange to hexane, yielding a final volume of 2 ml.

The subsequent analysis encompassed 16 PAHs and 10 aliphatic hydrocarbons. PAH compounds with 2–3 rings refer to those containing 2 or 3 benzene rings in their structure, while PAHs with 4–6 rings include compounds that have 4, 5, or 6 benzene rings within their chemical structure. The analytes are included as

- PAH 2–3 rings: naphthalene, acenaphthylene, acenaphthene, fluorene, phenanthrene, and anthracene
- PAH 4–6 rings: fluoranthene, pyrene, benz[a]anthracene, chrysene, benzo[o]fluoranthene, benzo[k]fluoranthene, benz(a)pyrene, indeno[1,2,3-cd] pyrene, dibenz[a,h]anthracene, and benzo[ghi]perylene
- Aliphatic hydrocarbons with 10 to 16 carbons: decane, undecane, dodecane, tridecane, tetradecane, pentadecane, and hexadecane
- Aliphatic hydrocarbons with 18 to 24 carbons: octadecane, nonadecane, eicosane, heneicosane, docosane, tricosane, and tetracosane.

Analysis was conducted through the application of gas chromatography coupled with a single quadrupole mass spectrometer (GC-MS/Agilent Technologies-GC 6890N) in Selective Ion Monitoring (SIM) mode, with decachlorobiphenyl utilized as an internal standard. External calibration standard solutions were employed for each target compound. Gas chromatographic separation was facilitated by a Varian VF5ms capillary column (30 m × 0.25 mm i.d., 0.25 μm film thickness). The injection mode was set to splitless, and the inlet temperature was maintained at 300°C. The oven temperature program involved a linear gradient: starting at 50°C, holding for 0.5 minutes, followed by a ramp of 25°C/min up to 290°C, and subsequently, a secondary ramp of 5°C/min up to 320°C, held for 5 minutes.

Quality control measures were implemented through duplicate analyses, calibration verification standards, clean matrix-spiked samples, and operational blanks, conducted for every batch of 10 samples. These procedures served to ensure the accuracy and precision of the analytical method. The mean recovery rates for the analytical surrogates were reported as follows: for PAHs, the recovery was 85 ± 20, for aliphatic, the recovery rate was at 114 ± 39%. The standard deviations reflect the variability in recovery efficiency across different samples. All employed organic solvents adhered to SupraSolv® Grade for GC-MS and were procured from Merck KGaA (Darmstadt, Germany). PAHs and aliphatic hydrocarbon standards, along with surrogate solutions, were acquired from Sigma-Aldrich.

### 2.5. Statistical analysis and results expression

Firstly, to compare the concentrations of organic pollutants in fiberbank substrates before and after the growth of each fungi species, a paired samples Students t-test (95% confidence level) was conducted. This allowed us to assess if there was a significant removal or degradation of targeted organic pollutants. Afterward, to evaluate the variations in pollutant removal percentages across the different fungal species, a one-way ANOVA test was utilized. This was followed by post-hoc pairwise comparisons using the LSD Test, maintaining a confidence level of 95%. Prior to these analyses, we ensured that the assumptions of normality, error independence, and equal variances were met. All these statistical procedures were conducted using the RStudio software, specifically version 4.1.1 of the R package.

## 3. Results and discussion

### 3.1. Growth percentage of WRF

**Figure 3.**
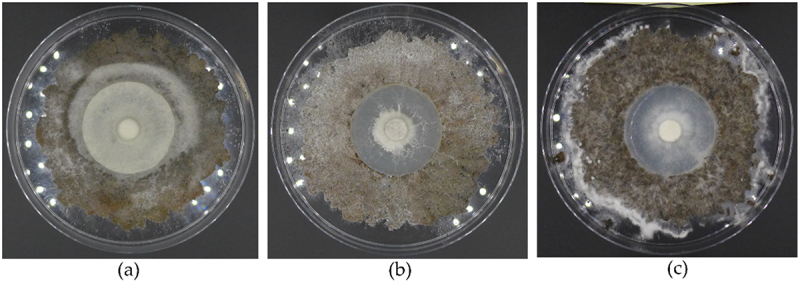
After two months of growth (a) *P. tremellosa*, (b) *P. gigantea*, (c) *D. crustulinus*. (photos: Burcu Hacıoğlu).

Fifteen species of WRF, namely, *B. adusta, C. muraii, D. crustulinus, G. applanatum, H. annosum, H. tabacina, L. sulphureus, P. ferrugineofuscus, P. punctatus, P. tremellosa, P. gigantea, S. odora, S. sanguinolentum, T. hirsuta*, and *T. ochracea*, showed to be able to grow on fiberbank-plates (Figure 3).

The growth percentages of the fungal mycelia are presented in Table 2. The fungal species *D. lindbladii*, *H. aurantiacus*, *I. benzoinum*, *J. collabens*, *J. luteoalba*, *M. taxicola*, *P. chrysoloma*, *P. nigrolimitatus*, *P. populicola*, *P. viticola*, and *S. rugosum* demonstrated limited growth, restricted to the 5 cm Hagem agar discs within the Petri dishes. These species showed no mycelial expansion onto the fiberbanks substrate. Therefore, in cases where mycelial expansion onto the fiberbanks material was absent, these were recorded as a growth value of zero in the study’s data set.

**Table 2.** The mean (*n* = 3) growth area percentages (%) of fungal mycelia in control (perlite) and fiberbanks treatments.

Species	Growth area (%)^a^		Tolerance Index^b^
	Fiberbanks (%)	Control (%)	
*B. adusta*	19.35 ± 4.69	30.71	0.63
*C. muraii*	32.23 ± 0.66	32.58	0.99
*D. crustulinus*	67.92 ± 5.3	0.00*	NA
*D. lindbladii*	0*	0.00*	NA
*G. applanatum*	41.11 ± 8.77	0.00*	NA
*H. aurantiacus*	0*	0.00*	NA
*H. annosum*	62.23 ± 4.82	0.00*	NA
*H. tabacina*	68.26 ± 2.71	60.5	1.13
*I. benzoinum*	0*	68.88	0
*J. collabens*	0*	30.68	0
*J. luteoalba*	0*	48.47	0
*L. sulphureus*	72.2 ± 2.18	55.52	1.3
*M. taxicola*	0*	0.00*	NA
*P. chrysoloma*	0*	0.00*	NA
*P. ferrugineofuscus*	60.46 ± 2.56	0.00*	NA
*P. nigrolimitatus*	0*	0.00*	NA
*P. populicola*	0*	27.28	0
*P. punctatus*	66.88 ± 1.03	50.15	1.33
*P. viticola*	0*	33.34	0
*P. tremellosa*	65.78 ± 0.1	57.69	1.14
*P. gigantea*	37.22 ± 1.68	21.38	1.74
*S. odora*	41.26 ± 8.24	20.79	1.98
*S. rugosum*	0*	64.52	0
*S. sanguinolentum*	65.78 ± 0.37	41.58	1.58
*T. hirsuta*	51.68 ± 2.02	58.85	0.88
*T. ochracea*	17.69 ± 1.06	55.87	0.32

^a^The average percentage of mycelial growth area relative to the total plate area is calculated using the formula: Growth area (%) = (Growth area of mycelium/Total area of the petri dish) x100 (The petri dish has a diameter of 16 cm). Values are the mean of three replicates *Instances of zero values are indicative of two scenarios: either a complete absence of mycelial growth or instances where growth is confined solely to the 5 cm-Hagem-agar, with no extension over the fiberbanks or perlite substrates.

^a^The Tolerance Index is calculated as: TI = Growth Area in fiberbanks (%)/Growth Area in Perlite (%).

NA: The TI value cannot be calculated since the growth area percentage in the control is zero.

The mycelial growth exhibited considerable variation across the different fungal species, as reflected in the calculated Tolerance Index (TI). TI values close to or above 1 indicate an attraction of the fungal species to the fiberbanks, while values below 1 indicate increasing growth inhibition as they approach zero [37].

Previous studies have indicated that the growth dynamics of fungi can be heavily influenced by the nutritional composition of the agar media. According to these findings, mycelial growth may sometimes be more noticeable in nutrient-limited media compared to nutrient-rich environments [38]. Specifically, slower-growing species, which have lower nutritional requirements, might prefer to limit their growth to the Hagem agar region. Contrarily, faster-growing species may extend beyond this region in search of additional nutrients, as the resources in the Hagem agar are insufficient to sustain their rapid development.

An alternative hypothesis is that some mycelial growth may halt upon reaching the fiberbank substrate due to its toxicity. The tolerance of WRF species to organic pollutants and heavy metals varies between species [39]. This variation in tolerance might explain the different growth patterns observed across various WRF species in the presence of fiberbank substrates.

*Diplomitoporus crustulinus*, *G. applanatum*, *H. annosum*, and *P. ferrugineofuscus* exhibited growth on fiberbanks material, while no growth was observed in the control samples, making it impossible to calculate TI values (Table 2). This suggests that these fungi utilize nutrients from the fiberbank material rather than relying solely on the Hagem agar for sustenance. Furthermore, their ability to thrive in the fiberbank material indicates their resilience to its inherent toxicity, suggesting that these species are well adapted to cope with the environmental conditions presented by the fiberbank substrate.

*Laetiporus sulphureus*, *S. odora*, and *S. sanguinolentum* exhibited higher growth in fiberbank material than in the control samples, indicating their tolerance to the material’s toxicity (TI values > 1; Table 2) and potential use of it as a nutrient source. These species also grew in control samples above perlite, suggesting an initial search for nutrients. However, in the control samples, mycelial growth ceased, likely due to perlite’s lack of nutritional value for fungi. In contrast, their mycelium extended further on fiberbank materials, underscoring their capability to utilize the fiberbank substrate’s components for growth, despite its toxic nature.

*Bjerkandera adusta* and *T. ochracea* exhibited growth in both fiberbanks and control samples, suggesting initial adaptability to both environments. However, their mycelial growth ceased in the fiberbank substrate, while it continued further in the control samples, possibly due to the toxicity in the fiberbanks material (TI < 1; Table 2). This behavior indicates that these species, likely among the faster-growing WRF, actively seek additional nutrients beyond what Hagem agar alone can provide. Upon encountering fiberbank material, their growth ceased, suggesting that the fiberbank environment poses a hostile setting for these species, likely due to toxic constituents that inhibit further growth.

*Ischnoderma benzoinum*, *J. collabens*, *J. luteoalba*, *P. populicola*, *P. viticola*, and *S. rugosum* exhibited growth exclusively in control samples, with no growth observed in fiberbanks material (TI = 0; Table 2). This pattern suggests that the toxicity of the fiberbanks substrate inhibits the growth of these fungal species, indicating their inability to withstand unfavorable conditions within the fiberbanks environment. In the control samples, their extended growth beyond the Hagem agar medium indicates a search for additional nutrients.

*Cystostereum muraii*, *H. tabacina*, *P. punctatus*, *P. tremellosa*, *P. gigantea*, and *T. hirsuta* showed similar growth in both the fiberbank and control environments, with TI values close to 1 (Table 2). This suggests that these species are resilient to the toxic effects of the fiberbank material. Their growth is likely influenced more by their natural growth rates and metabolic abilities, indicating they might have an inherent ability to handle or neutralize the toxins in the fiberbanks material.

*Diplomitoporus lindbladii*, *H. aurantiacus*, *M. taxicola*, *P. chrysoloma*, and *P. nigrolimitatus* displayed no growth in either fiberbank material or control samples, and thus TI values could not be calculated. This behavior can be attributed to the inherently slower growth rates of some fungal species, which do not actively seek out new nutrient sources as quickly as their faster-growing counterparts. In this scenario, it is plausible to suggest that these fungi are of the slow-growing variety, finding the nutrients present in the Hagem agar medium sufficient for their needs over the two-month period.

*Laetiporus sulphureus*, *H. tabacina*, *D. crustulinus*, *P. punctatus*, *S. sanguinolentum*, *P. tremellosa*, *H. annosum*, and *P. ferrugineofuscus* exhibited the highest growth area percentages among the 26 WRF species. These data suggest that these eight WRF species possess a tolerance to the toxicity of fiberbank material, potentially utilizing the fiberbank substrate as a nutritional source.

### 3.2. Removal percentage of organic pollutants

Only the WRF species that exhibited growth in fiberbank material were analyzed for their capacity to degrade organic pollutants. Species such as *D. lindbladii*, *H. aurantiacus*, *I. benzoinum*, *J. collabens*, *J. luteoalba*, *M. taxicola*, *P. chrysoloma*, *P. nigrolimitatus*, *P. populicola*, *P. viticola*, and *S. rugosum*, which did not show any growth, were not included in further degradation studies. This approach ensures that the focus remains on evaluating the bioremediation potential of fungi that can adapt to and utilize nutrients within the fiberbank material.

A paired samples Student’s t test showed that the degradation of aliphatic hydrocarbons by the fungi did not yield many significant results. For LMW aliphatics (C10–16), only *D. crustulinus* and *G. applanatum* showed a significant reduction in initial and final concentrations. For higher molecular weight aliphatics (C18–24), initial concentrations were too low to measure accurately (these results are shared in the supplementary material, Table SM-4). Therefore, the main text focuses on the degradation of PAHs. Detailed data on all analytes’ concentrations for each species can be found in the supplementary material, specifically in Table SM-3.

**Figure 4.**
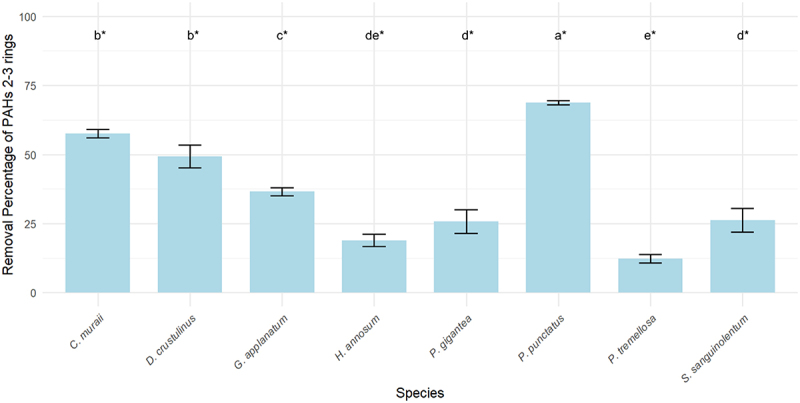
Removal percentage of 2–3 rings PAHs by WRF with standard deviation. (*) the letters indicate the outcomes derived from the post-hoc LSD test.

Figure 4 shows the removal efficiencies of PAHs with 2–3 ring structures by the tested WRF species. *C. muraii*, *D. crustulinus*, *G. applanatum*, *H. annosum*, *P. gigantea*, *P. punctatus*, *P. tremellosa*, and *S. sanguinolentum* significantly removed 2–3 ring PAHs. *P. punctatus* had the highest removal efficiency at 68.8%, followed by *C. muraii* at 57.6% and *D. crustulinus* at 49.3%. These results are notable compared to other studies. For example, *Pleurotus ostreatus* removed 41–64% of phenanthrene over two months [40], and *Anthracophyllum discolor* removed 62% of phenanthrene and 73% of anthracene within 60 days [41].

While some studies report higher degradation percentages for LMW PAHs, such as *Peniophora incarnata* achieving 95.3% removal of phenanthrene after two weeks of incubation, *Phlebia brevispora* reaching an 80.4% removal rate of anthracene [42], and *Phanerochaete sordida* removing 89.2% of phenanthrene after 32 days with surfactants [43], there is no assurance that these fungi can tolerate the toxicity of the fiberbank substrate. For instance, *B. adusta* is known for its ability to degrade PAHs [19], but it showed limited growth on fiberbank material and did not significantly degrade LMW PAHs. This could be due to other contaminants, such as PTEs, and the complexity of the substrate.

**Figure 5.**
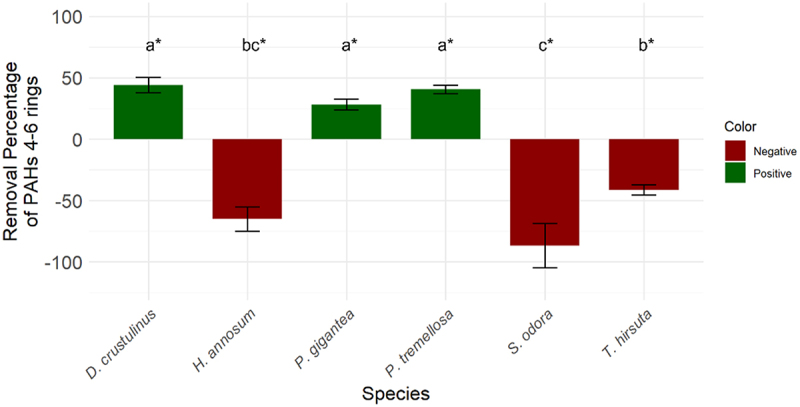
The removal percentage of 4–6 ring PAHs, with standard deviation, by WRF is represented in a color-coded format. Green indicates a positive removal effect, where the concentration of 4–6 ring PAHs post-treatment by the fungi is lower than the initial concentration. Conversely, red signifies an increase, indicating that the concentration of 4–6 ring PAHs post-treatment is higher than the initial concentration. This color coding distinguishes between the efficacy of different fungi species in reducing (as desired) or increasing the concentration of these specific PAHs. (*) the letters indicate the outcomes derived from the post-hoc LSD test.

Figure 5 illustrates the significant removal percentage of 4–6 ring PAHs by various fungi species. Notably, *D. crustulinus*, *P. tremellosa*, and *P. gigantea* have demonstrated significant removal of these highly recalcitrant pollutants. Specifically, *D. crustulinus* achieved a 44% removal of HMW PAHs, and *P. tremellosa* achieved a 40% removal, highlighting their efficient performance. These degradation rates align closely with contemporary studies. For example, *Phlebia brevispora* achieved 65% and 63% removal of pyrene and 12% and 8% of benzo(a)pyrene in 15 days [44]. Similarly, *Phanerochaete sordida* removed 61.9% of pyrene and 28.6% of benzo(a)pyrene in 32 days [43]. Notably, *D. crustulinus* and *P. tremellosa* have demonstrated not only the ability to break down HMW PAHs effectively but also considerable resistance to the toxicity of the fiberbank material.

The data indicate that *D. crustulinus* and *P. tremellosa* could be promising candidates for bioremediation strategies. They are particularly effective in tolerating fiberbank contaminants and remediating PAHs with 4–6 rings. According to the literature, some white-rot fungi species have shown significant contaminant removal capacities. For example, *Anthracophyllum discolor* can remove 54% of fluoranthene, 60% of pyrene, and 75% of benzo(a)pyrene in 28 days of inoculation in liquid media [41]. Meanwhile, *Pleurotus eryngii* has demonstrated a 59% removal capacity of benzo[a]pyrene in static liquid culture over 20 days [45]. Achieving higher removal percentages may be challenging due to the complexity of fiberbank materials. These HMW PAHs in fiberbanks show low bioavailability due to their strong adsorption onto organic matter [46,47]. The underwater aging process also makes them more resistant to biodegradation efforts. This emphasizes the challenges posed by both the environmental matrix and the physicochemical properties of the contaminants in the bioremediation of fiberbanks.

Interestingly, *H. annosum*, *S. odora*, and *T. hirsuta* exhibited an increase in 4–6 ring PAHs. This implies that the concentration of these PAHs in the fiberbank substrate was higher after fungal treatment compared to the initial concentrations. Several factors could contribute to this outcome. Firstly, the inherent complexity of the fiberbank material poses a significant challenge in achieving uniform homogenization. This heterogeneity could affect the interaction between the fungi mycelia and the PAHs, leading to inconsistent results.

Another explanation could be related to the extracellular enzyme activities of WRF. These fungi can secrete enzymes into their environment, degrading compounds and converting them into others, potentially including PAH-like compounds. These compounds might be similar in structure to PAHs, making it difficult to differentiate them from the target PAHs using the available analytical methods. As a result, the measured concentration of 4–6 ring PAHs post-treatment could be artificially inflated, reflecting the presence of these fungal by-products rather than an actual increase in the PAHs initially present in the fiberbank material. Moreover, these enzymatic activities could increase the bioavailability of HMW PAHs, influencing the outcomes of analytical assessments [46,47].

The experiment, limited to a two-month duration, only measured the PAH concentrations in the fiberbank material at the beginning and end of the treatment period. This limitation suggests that variations in degradation rates may have occurred at intermediate stages, and potentially higher degradation levels could be achieved if the experiments were extended beyond two months. To fully understand the dynamics of PAH degradation over time under native WRF treatment, more comprehensive research is required. Such studies should include regular monitoring of PAH levels throughout the treatment process to accurately evaluate the degradation capabilities of WRF and avoid prematurely discounting their potential for organic pollutant remediation.

## 4. Conclusions

The remediation of fiberbanks presents a complex challenge due to the insufficient understanding of their complicated characteristics. Unlike typical polluted soils or sediments, fiberbanks and fiber-rich sediments contain significant amounts of organic matter at various stages of decomposition, giving them unique and changing physical and chemical properties. This complexity is further heightened by the presence of PTEs and a variety of organic pollutants, all of which have aged underwater, resulting in highly variable contamination levels and patterns across different locations. Effective remediation strategies must consider the initial concentrations and bioavailability of these organic pollutants, highlighting the need for broader and more detailed experimental methodologies.

Addressing this need, a comparative study was conducted to assess the biodegradation capabilities of 26 native white rot fungi (WRF) species. This study identified three species—*D. crustulinus*, *P. tremellosa*, and *P. gigantea*—as particularly effective for large-scale bioremediation experiments, especially in degrading HMW PAHs. Notably, *D. crustulinus* demonstrated superior performance, marked by a high percentage of PAH removal for both low and HMW compounds, along with significant mycelial growth. These results emphasize *D. crustulinus* as a strong candidate for the bioremediation of organic pollutants, underscoring its potential in environmental remediation applications.

## Supplementary Material

Unlocking the biodegradative potential.docx

## Data Availability

The authors confirm that the data supporting the findings of this study are available within the article [and/or] its supplementary materials.
