# Supplementary material for: Unlocking the biodegradative potential of native white-rot fungi: a comparative study of fiberbank organic pollutant mycoremediation
Source: Bioengineered. 2024 Sep 2;15(1):2396642. doi: 10.1080/21655979.2024.2396642 (PMC11370975; doi:10.1080/21655979.2024.2396642)
Supplement: Unlocking the biodegradative potential.docx [file KBIE_A_2396642_SM4204.docx]

Unlocking the biodegradative potential of native white-rot fungi: A comparative study on fiberbanks organic pollutants mycoremediation

Burcu Hacioglu ^1^, Gabriel Dupaul ^1^, Gabriela Paladino ^1^, Mattias Edman ^1^ and Erik Hedenström ^1^

^1^Mid Sweden University, Department of Natural Science, Design and Sustainable Development

**Table SM-1.** Dewatered fresh (before autoclave) fiberbanks analysis report from accredited external laboratory.

| **Analyte** | **Result** | **Unit** | **Measurement uncertainty** | **Metod/ref** |  |
| --- | --- | --- | --- | --- | --- |
| Torrsubstans | 0.96 | % | 5% | SS-EN 12880:2000 | b) |
| Torrsubstans | 0.97 | % | 5% | SS-EN 12880:2000 | a) |
| Alifater >C8-C10 | < 17 | mg/kg Ts | 35% | SPI 2011 | b)* |
| Alifater >C10-C12 | < 17 | mg/kg Ts | 30% | SPI 2011 | b)* |
| Alifater >C12-C16 | < 17 | mg/kg Ts | 30% | SPI 2011 | b)* |
| Alifater >C16-C35 | 2100 | mg/kg Ts | 30% | SPI 2011 | b)* |
| Aromater >C8-C10 | < 34 | mg/kg Ts | 30% | SPI 2011 | b)* |
| Aromater >C10-C16 | < 3.4 | mg/kg Ts | 20% | SPI 2011 | b)* |
| Aromater >C16-C35 | 1.7 | mg/kg Ts | 25% | SPI 2011 | b)* |
| Metylpyren/fluorantener | < 1.7 | mg/kg Ts | 25% | SPI 2011 | b)* |
| Metylkrysener/benzo(a)antracener | < 1.7 | mg/kg Ts | 25% | SPI 2011 | b)* |
| Bens(a)antracen | < 0.12 | mg/kg Ts | 25% | ISO 18287:2008 mod | b) |
| Krysen | < 0.12 | mg/kg Ts | 25% | ISO 18287:2008 mod | b) |
| Benso(b,k)fluoranten | 0.18 | mg/kg Ts | 25% | ISO 18287:2008 mod | b) |
| Benso(a)pyren | < 0.12 | mg/kg Ts | 25% | ISO 18287:2008 mod | b) |
| Indeno(1,2,3-cd)pyren | < 0.12 | mg/kg Ts | 25% | ISO 18287:2008 mod | b) |
| Dibens(a,h)antracen | < 0.12 | mg/kg Ts | 30% | ISO 18287:2008 mod | b) |
| Naftalen | 2.8 | mg/kg Ts | 25% | ISO 18287:2008 mod | b) |
| Acenaftylen | 0.18 | mg/kg Ts | 40% | ISO 18287:2008 mod | b) |
| Acenaften | 0.37 | mg/kg Ts | 25% | ISO 18287:2008 mod | b) |
| Fluoren | 0.13 | mg/kg Ts | 30% | ISO 18287:2008 mod | b) |
| Fenantren | 0.47 | mg/kg Ts | 25% | ISO 18287:2008 mod | b) |
| Antracen | < 0.12 | mg/kg Ts | 25% | ISO 18287:2008 mod | b) |
| Fluoranten | 0.31 | mg/kg Ts | 25% | ISO 18287:2008 mod | b) |
| Pyren | 0.44 | mg/kg Ts | 25% | ISO 18287:2008 mod | b) |
| Benso(g,h,i)perylen | < 0.12 | mg/kg Ts | 25% | ISO 18287:2008 mod | b) |
| Summa PAH med låg molekylvikt | 3.4 | mg/kg Ts |  |  | b) |
| Summa PAH med medelhög molekylvikt | 1.4 | mg/kg Ts |  |  | b) |
| Summa PAH med hög molekylvikt | 0.54 | mg/kg Ts |  |  | b) |
| Summa cancerogena PAH | 0.48 | mg/kg Ts |  |  | b) |
| Summa övriga PAH | 4.8 | mg/kg Ts |  |  | b) |
| Summa totala PAH16 | 5.3 | mg/kg Ts |  |  | b) |
| 1,1,1,2-Tetrakloretan | < 0.0050 | mg/kg Ts | 20% | EPA 5021 | b) |
| 1,1,1-Trikloretan | < 0.0050 | mg/kg Ts | 25% | EPA 5021 | b) |
| 1,1,2-Trikloretan | < 0.0050 | mg/kg Ts | 30% | EPA 5021 | b) |
| 1,1,2-Trikloreten | < 0.0050 | mg/kg Ts | 20% | EPA 5021 | b) |
| 1,1-Dikloretan | < 0.0050 | mg/kg Ts | 30% | EPA 5021 | b) |
| 1,1-Dikloreten | < 0.0050 | mg/kg Ts | 30% | EPA 5021 | b) |
| 1,1-Diklorpropen | < 0.0050 | mg/kg Ts | 25% | EPA 5021 | b) |
| 1,2,3-Triklorbensen | < 0.0050 | mg/kg Ts | 30% | EPA 5021 | b) |
| 1,2,3-Triklorpropan | < 0.0050 | mg/kg Ts | 25% | EPA 5021 | b) |
| 1,2,4-Triklorbensen | < 0.0050 | mg/kg Ts | 20% | EPA 5021 | b) |
| 1,2,4-Trimetylbensen | < 0.0050 | mg/kg Ts | 30% | EPA 5021 | b) |
| 1,2-Dibrometan | < 0.0050 | mg/kg Ts | 25% | EPA 5021 | b) |
| 1,2-Diklorbensen | < 0.0050 | mg/kg Ts | 15% | EPA 5021 | b) |
| 1,2-Dikloretan | < 0.0050 | mg/kg Ts | 25% | EPA 5021 | b) |
| 1,2-Diklorpropan | < 0.0050 | mg/kg Ts | 20% | EPA 5021 | b) |
| 1,3,5-Trimetylbensen | < 0.0050 | mg/kg Ts | 30% | EPA 5021 | b) |
| 1,3-Diklorbensen | < 0.0050 | mg/kg Ts | 15% | EPA 5021 | b) |
| 1,3-Diklorpropan | < 0.0050 | mg/kg Ts | 25% | EPA 5021 | b) |
| 1,3-Diklorpropen | < 0.0050 | mg/kg Ts | 25% | EPA 5021 | b) |
| 1,4-Diklorbensen | < 0.0050 | mg/kg Ts | 15% | EPA 5021 | b) |
| 2,2-Diklorpropan | < 0.0050 | mg/kg Ts | 30% | EPA 5021 | b) |
| 2-Klortoluen | < 0.0050 | mg/kg Ts | 30% | EPA 5021 | b) |
| 4-Klortoluen | < 0.0050 | mg/kg Ts | 30% | EPA 5021 | b) |
| Bensen | 0.91 | mg/kg Ts | 25% | EPA 5021 | b) |
| Brombensen | < 0.0050 | mg/kg Ts | 20% | EPA 5021 | b) |
| Bromdiklormetan | < 0.0050 | mg/kg Ts | 25% | EPA 5021 | b) |
| Bromklormetan | < 0.0050 | mg/kg Ts | 30% | EPA 5021 | b) |
| cis-1,2-Dikloreten | < 0.0050 | mg/kg Ts | 30% | EPA 5021 | b) |
| Dibromklormetan | < 0.0050 | mg/kg Ts | 25% | EPA 5021 | b) |
| Dibrommetan | < 0.0050 | mg/kg Ts | 30% | EPA 5021 | b) |
| Diklormetan | < 0.0050 | mg/kg Ts | 30% | EPA 5021 | b) |
| Etylbensen | 0.011 | mg/kg Ts | 20% | EPA 5021 | b) |
| Hexaklorbutadien (HCBD) | < 0.0050 | mg/kg Ts | 30% | EPA 5021 | b) |
| iso-Propylbensen | < 0.0050 | mg/kg Ts | 30% | EPA 5021 | b) |
| Klorbensen | < 0.0050 | mg/kg Ts | 25% | EPA 5021 | b) |
| m/p-Xylen | 0.013 | mg/kg Ts | 30% | EPA 5021 | b) |
| n-Butylbensen | < 0.0050 | mg/kg Ts | 30% | EPA 5021 | b) |
| o-Xylen | 0.0051 | mg/kg Ts | 30% | EPA 5021 | b) |
| p-Isopropyltoluen | 0.014 | mg/kg Ts | 30% | EPA 5021 | b) |
| Propylbensen | < 0.0050 | mg/kg Ts | 25% | EPA 5021 | b) |
| sec-Butylbensen | < 0.0050 | mg/kg Ts | 30% | EPA 5021 | b) |
| tert-Butylbensen | < 0.0050 | mg/kg Ts | 30% | EPA 5021 | b) |
| Tetrakloreten | < 0.0050 | mg/kg Ts | 20% | EPA 5021 | b) |
| Tetraklormetan | < 0.0050 | mg/kg Ts | 25% | EPA 5021 | b) |
| Toluen | 0.35 | mg/kg Ts | 20% | EPA 5021 | b) |
| trans-1,2-Dikloreten | < 0.0050 | mg/kg Ts | 30% | EPA 5021 | b) |
| trans-1,3-Diklorpropen | < 0.0050 | mg/kg Ts | 25% | EPA 5021 | b) |
| Tribrommetan | < 0.0050 | mg/kg Ts | 30% | EPA 5021 | b) |
| Triklorflourmetan (CFC-11) | < 0.0050 | mg/kg Ts | 30% | EPA 5021 | b) |
| Triklormetan | < 0.0050 | mg/kg Ts | 25% | EPA 5021 | b) |
| Aluminum Al | 940 | mg/kg Ts | 15% | SS028311 / ICP-AES | b) |
| Arsenik As | < 9.4 | mg/kg Ts | 25% | EN ISO 11885:2009 / SS 028311 utg 1 | b)* |
| Barium Ba | 16 | mg/kg Ts | 25% | EN ISO 11885:2009 / SS 028311 utg 1 | b)* |
| Bly Pb | 6.5 | mg/kg Ts | 25% | EN ISO 11885:2009 / SS 028311 utg 1 | b)* |
| Kadmium Cd | < 0.52 | mg/kg Ts | 25% | EN ISO 11885:2009 / SS 028311 utg 1 | b)* |
| Kobolt Co | < 2.4 | mg/kg Ts | 25% | EN ISO 11885:2009 / SS 028311 utg 1 | b)* |
| Koppar Cu | 22 | mg/kg Ts | 25% | EN ISO 11885:2009 / SS 028311 utg 1 | b)* |
| Krom Cr | 5 | mg/kg Ts | 25% | EN ISO 11885:2009 / SS 028311 utg 1 | b)* |
| Kvicksilver Hg | 0.049 | mg/kg Ts | 20% | SS028311mod/SS-EN ISO17852mod | b)* |
| Nickel Ni | 3 | mg/kg Ts | 25% | EN ISO 11885:2009 / SS 028311 utg 1 | b)* |
| Silver Ag | < 0.94 | mg/kg Ts | 25% | EN ISO 17294-2:2016 / SS 028311, utg 1 | b)* |
| Tenn Sn | 1.1 | mg/kg Ts | 25% | EN ISO 17294-2:2016 / SS 028311, utg 1 | b)* |
| Vanadin V | < 9.4 | mg/kg Ts | 25% | EN ISO 11885:2009 / SS 028311 utg 1 | b)* |
| Zink Zn | 67 | mg/kg Ts | 25% | EN ISO 11885:2009 / SS 028311 utg 1 | b)* |
| Summa Diklorfenoler | < 1.0 | mg/kg Ts | 20% | Internal Method LidMiljö.0A.01.17 | b)* |
| Summa Triklorfenol | < 1.0 | mg/kg Ts | 20% | Internal Method LidMiljö.0A.01.17 | b)* |
| Summa Tetraklorfenol | < 1.0 | mg/kg Ts | 20% | Internal Method LidMiljö.0A.01.17 | b)* |
| Pentaklorfenol | < 1.0 | mg/kg Ts | 20% | Internal Method LidMiljö.0A.01.17 | b)* |
| DDT-o,p | < 0.10 | mg/kg Ts | 20% | Internal Method LidMiljö.0A.01.17 | b)* |
| DDT,p,p'- | < 0.10 | mg/kg Ts | 20% | Internal Method LidMiljö.0A.01.17 | b)* |
| DDE,o,p- | < 0.10 | mg/kg Ts | 20% | Internal Method LidMiljö.0A.01.17 | b)* |
| DDE-p,p | < 0.10 | mg/kg Ts | 20% | Internal Method LidMiljö.0A.01.17 | b)* |
| HCH-alfa | < 0.10 | mg/kg Ts | 20% | Internal Method LidMiljö.0A.01.17 | b)* |
| HCH-beta | < 0.10 | mg/kg Ts | 20% | Internal Method LidMiljö.0A.01.17 | b)* |
| HCH-delta | < 0.10 | mg/kg Ts | 20% | Internal Method LidMiljö.0A.01.17 | b)* |
| HCH,gamma- (Lindane) | < 0.10 | mg/kg Ts | 20% | Internal Method LidMiljö.0A.01.17 | b)* |
| Hexaklorbensen | < 0.10 | mg/kg Ts | 20% | Internal Method LidMiljö.0A.01.17 | b)* |
| Endosulfan-alpha | < 0.10 | mg/kg Ts | 20% | Internal Method LidMiljö.0A.01.17 | b)* |
| Endosulfan-beta | < 0.10 | mg/kg Ts | 20% | Internal Method LidMiljö.0A.01.17 | b)* |
| Endosulfan-sulfate | < 0.10 | mg/kg Ts | 20% | Internal Method LidMiljö.0A.01.17 | b)* |
| Dieldrin | < 0.10 | mg/kg Ts | 20% | Internal Method LidMiljö.0A.01.17 | b)* |
| Endrin | < 0.10 | mg/kg Ts | 20% | Internal Method LidMiljö.0A.01.17 | b)* |
| PCB 28 | < 0.10 | mg/kg Ts | 20% | Internal Method LidMiljö.0A.01.17 | b)* |
| PCB 52 | < 0.10 | mg/kg Ts | 20% | Internal Method LidMiljö.0A.01.17 | b)* |
| PCB 101 | < 0.10 | mg/kg Ts | 20% | Internal Method LidMiljö.0A.01.17 | b)* |
| PCB 118 | < 0.10 | mg/kg Ts | 20% | Internal Method LidMiljö.0A.01.17 | b)* |
| PCB 153 | < 0.10 | mg/kg Ts | 20% | Internal Method LidMiljö.0A.01.17 | b)* |
| PCB 138 | < 0.10 | mg/kg Ts | 20% | Internal Method LidMiljö.0A.01.17 | b)* |
| PCB 180 | < 0.10 | mg/kg Ts | 20% | Internal Method LidMiljö.0A.01.17 | b)* |
| S:a PCB (7st) | < 0.40 | mg/kg Ts |  | Internal Method LidMiljö.0A.01.17 | b)* |
| N-nitroso-di-n-propylamin | < 0.10 | mg/kg Ts | 20% | Internal Method LidMiljö.0A.01.17 | b)* |
| Nitrobensen | < 0.10 | mg/kg Ts | 20% | Internal Method LidMiljö.0A.01.17 | b)* |
| Azobensen | < 0.10 | mg/kg Ts | 20% | Internal Method LidMiljö.0A.01.17 | b)* |
| N-nitrosodifenylamin | < 0.10 | mg/kg Ts | 20% | Internal Method LidMiljö.0A.01.17 | b)* |
| 2,6-Dinitrotoluen | < 0.10 | mg/kg Ts | 20% | Internal Method LidMiljö.0A.01.17 | b)* |
| 2,4-Dinitrotoluen | < 0.10 | mg/kg Ts | 20% | Internal Method LidMiljö.0A.01.17 | b)* |
| Bis(2-kloretyl)eter | < 0.10 | mg/kg Ts | 20% | Internal Method LidMiljö.0A.01.17 | b)* |
| Bis(2-kloroisopropyl)eter | < 0.10 | mg/kg Ts | 20% | Internal Method LidMiljö.0A.01.17 | b)* |
| Hexakloretan | < 0.10 | mg/kg Ts | 20% | Internal Method LidMiljö.0A.01.17 | b)* |
| Isophorone | < 0.10 | mg/kg Ts | 20% | Internal Method LidMiljö.0A.01.17 | b)* |
| Bis(2-kloretoxy)metan | < 0.10 | mg/kg Ts | 20% | Internal Method LidMiljö.0A.01.17 | b)* |
| 2-Klornaftalen | < 0.10 | mg/kg Ts | 20% | Internal Method LidMiljö.0A.01.17 | b)* |
| 4-Klorfenyl fenyleter | < 0.10 | mg/kg Ts | 20% | Internal Method LidMiljö.0A.01.17 | b)* |
| 4-Bromofenyl fenyleter | < 0.10 | mg/kg Ts | 20% | Internal Method LidMiljö.0A.01.17 | b)* |
| Pentaklorbensen | < 0.10 | mg/kg Ts | 20% | Internal Method LidMiljö.0A.01.17 | b)* |
| Dimetylftalat (DMP) | < 0.10 | mg/kg Ts | 20% | Internal Method LidMiljö.0A.01.17 | b)* |
| Dietylftalat | < 0.10 | mg/kg Ts | 20% | Internal Method LidMiljö.0A.01.17 | b)* |
| Di-n-butylftalat | < 0.10 | mg/kg Ts | 20% | Internal Method LidMiljö.0A.01.17 | b)* |
| Bensylbutylftalat | < 0.10 | mg/kg Ts | 20% | Internal Method LidMiljö.0A.01.17 | b)* |
| Di-(2-etylhexyl)ftalat | < 1.0 | mg/kg Ts | 20% | Internal Method LidMiljö.0A.01.17 | b)* |
| Di-n-oktylftalat | < 0.10 | mg/kg Ts | 20% | Internal Method LidMiljö.0A.01.17 | b)* |
| Metylkvicksilver | 0.83 | µg/kg Ts |  | EPA Method 1630 | c)* |
| 1-(3,4-Dichlorophenyl)-3-methylurea | <1.0 | µg/kg Ts | 27% | J. of Chromatogr. A, 1217 (2010) 2933–2939 mod. | a) |
| 1-(3,4-Dichlorophenyl)urea | <1.0 | µg/kg Ts | 27% | J. of Chromatogr. A, 1217 (2010) 2933–2939 mod. | a) |
| 2,4,5-T | <10 | µg/kg Ts | 27% | J. of Chromatogr. A, 1217 (2010) 2933–2939 mod. | a) |
| 2,4-D | <10 | µg/kg Ts | 27% | J. of Chromatogr. A, 1217 (2010) 2933–2939 mod. | a) |
| 2,4-Dichlorprop | <10 | µg/kg Ts | 27% | J. of Chromatogr. A, 1217 (2010) 2933–2939 mod. | a) |
| 2,6-Dichlorobenzamide | <10 | µg/kg Ts | 27% | J. of Chromatogr. A, 1217 (2010) 2933–2939 mod. | a) |
| Atrazine | <10 | µg/kg Ts | 27% | J. of Chromatogr. A, 1217 (2010) 2933–2939 mod. | a) |
| Atrazine-desethyl | <10 | µg/kg Ts | 27% | J. of Chromatogr. A, 1217 (2010) 2933–2939 mod. | a) |
| Atrazine-desisopropyl | <10 | µg/kg Ts | 27% | J. of Chromatogr. A, 1217 (2010) 2933–2939 mod. | a) |
| Bentazone | <10 | µg/kg Ts | 27% | J. of Chromatogr. A, 1217 (2010) 2933–2939 mod. | a) |
| Cyanazine | <10 | µg/kg Ts | 27% | J. of Chromatogr. A, 1217 (2010) 2933–2939 mod. | a) |
| Diuron | <1.0 | µg/kg Ts | 27% | J. of Chromatogr. A, 1217 (2010) 2933–2939 mod. | a) |
| Imazapyr | <10 | µg/kg Ts | 27% | J. of Chromatogr. A, 1217 (2010) 2933–2939 mod. | a) |
| Linuron | <10 | µg/kg Ts | 27% | J. of Chromatogr. A, 1217 (2010) 2933–2939 mod. | a) |
| MCPA | <10 | µg/kg Ts | 27% | J. of Chromatogr. A, 1217 (2010) 2933–2939 mod. | a) |
| Mecoprop | <10 | µg/kg Ts | 27% | J. of Chromatogr. A, 1217 (2010) 2933–2939 mod. | a) |
| Simazine | <10 | µg/kg Ts | 27% | J. of Chromatogr. A, 1217 (2010) 2933–2939 mod. | a) |
| Terbuthylazine | <10 | µg/kg Ts | 27% | J. of Chromatogr. A, 1217 (2010) 2933–2939 mod. | a) |

Non-accredited analyzes are marked with *

The measurement uncertainty, unless otherwise stated, is reported as extended measurement uncertainty with a coverage factor of 2. Increased reporting limit for PAHs, aliphatics and aromatics due to difficult sample matrix.

1. Eurofins Food & Feed Testing Sweden (Lidköping), SWEDEN, ISO/IEC 17025:2017 SWEDAC 1977
2. Eurofins Environment Testing Sweden AB, SWEDEN, ISO/IEC 17025:2017 SWEDAC 1125
3. IVL Svenska Miljöinstitutet AB, SWEDEN

**Table SM-2.** Dewatered autoclaved fiberbanks analysis report from accredited external laboratory. This fiberbanks used as a growing material.

| Analyte | Result | Unit | Measurement uncertainty | Metod/ref |  |
| --- | --- | --- | --- | --- | --- |
| Torrsubstans | 96.1 | % | 5% | SS-EN 12880:2000 | b) |
| Alifater >C8-C10 | < 20 | mg/kg Ts | 35% | SPI 2011 | b)* |
| Alifater >C10-C12 | < 20 | mg/kg Ts | 30% | SPI 2011 | b)* |
| Alifater >C12-C16 | < 20 | mg/kg Ts | 30% | SPI 2011 | b)* |
| Alifater >C16-C35 | 3000 | mg/kg Ts | 30% | SPI 2011 | b)* |
| Aromater >C8-C10 | < 40 | mg/kg Ts | 30% | SPI 2011 | b)* |
| Aromater >C10-C16 | < 4.0 | mg/kg Ts | 20% | SPI 2011 | b)* |
| Aromater >C16-C35 | 2 | mg/kg Ts | 25% | SPI 2011 | b)* |
| Metylpyren/fluorantener | < 2.0 | mg/kg Ts | 25% | SPI 2011 | b)* |
| Metylkrysener/benzo(a)antracener | < 2.0 | mg/kg Ts | 25% | SPI 2011 | b)* |
| Bens(a)antracen | < 0.14 | mg/kg Ts | 25% | ISO 18287:2008 mod | b) |
| Krysen | < 0.14 | mg/kg Ts | 25% | ISO 18287:2008 mod | b) |
| Benso(b,k)fluoranten | < 0.14 | mg/kg Ts | 25% | ISO 18287:2008 mod | b) |
| Benso(a)pyren | < 0.14 | mg/kg Ts | 25% | ISO 18287:2008 mod | b) |
| Indeno(1,2,3-cd)pyren | < 0.14 | mg/kg Ts | 25% | ISO 18287:2008 mod | b) |
| Dibens(a,h)antracen | < 0.14 | mg/kg Ts | 30% | ISO 18287:2008 mod | b) |
| Naftalen | 2.3 | mg/kg Ts | 25% | ISO 18287:2008 mod | b) |
| Acenaftylen | 0.17 | mg/kg Ts | 40% | ISO 18287:2008 mod | b) |
| Acenaften | 0.39 | mg/kg Ts | 25% | ISO 18287:2008 mod | b) |
| Fluoren | 0.14 | mg/kg Ts | 30% | ISO 18287:2008 mod | b) |
| Fenantren | 0.37 | mg/kg Ts | 25% | ISO 18287:2008 mod | b) |
| Antracen | < 0.14 | mg/kg Ts | 25% | ISO 18287:2008 mod | b) |
| Fluoranten | 0.2 | mg/kg Ts | 25% | ISO 18287:2008 mod | b) |
| Pyren | 0.31 | mg/kg Ts | 25% | ISO 18287:2008 mod | b) |
| Benso(g,h,i)perylen | < 0.14 | mg/kg Ts | 25% | ISO 18287:2008 mod | b) |
| Summa PAH med låg molekylvikt | 2.9 | mg/kg Ts |  |  | b) |
| Summa PAH med medelhög molekylvikt | 1.1 | mg/kg Ts |  |  | b) |
| Summa PAH med hög molekylvikt | < 0.49 | mg/kg Ts |  |  | b) |
| Summa cancerogena PAH | < 0.42 | mg/kg Ts |  |  | b) |
| Summa övriga PAH | 4 | mg/kg Ts |  |  | b) |
| Summa totala PAH16 | 4.4 | mg/kg Ts |  |  | b) |
| 1,1,1,2-Tetrakloretan | < 0.0050 | mg/kg Ts | 20% | EPA 5021 | b) |
| 1,1,1-Trikloretan | < 0.0050 | mg/kg Ts | 25% | EPA 5021 | b) |
| 1,1,2-Trikloretan | < 0.0050 | mg/kg Ts | 30% | EPA 5021 | b) |
| 1,1,2-Trikloreten | < 0.0050 | mg/kg Ts | 20% | EPA 5021 | b) |
| 1,1-Dikloretan | < 0.0050 | mg/kg Ts | 30% | EPA 5021 | b) |
| 1,1-Dikloreten | < 0.0050 | mg/kg Ts | 30% | EPA 5021 | b) |
| 1,1-Diklorpropen | < 0.0050 | mg/kg Ts | 25% | EPA 5021 | b) |
| 1,2,3-Triklorbensen | < 0.0050 | mg/kg Ts | 30% | EPA 5021 | b) |
| 1,2,3-Triklorpropan | < 0.0050 | mg/kg Ts | 25% | EPA 5021 | b) |
| 1,2,4-Triklorbensen | < 0.0050 | mg/kg Ts | 20% | EPA 5021 | b) |
| 1,2,4-Trimetylbensen | < 0.0050 | mg/kg Ts | 30% | EPA 5021 | b) |
| 1,2-Dibrometan | < 0.0050 | mg/kg Ts | 25% | EPA 5021 | b) |
| 1,2-Diklorbensen | < 0.0050 | mg/kg Ts | 15% | EPA 5021 | b) |
| 1,2-Dikloretan | < 0.0050 | mg/kg Ts | 25% | EPA 5021 | b) |
| 1,2-Diklorpropan | < 0.0050 | mg/kg Ts | 20% | EPA 5021 | b) |
| 1,3,5-Trimetylbensen | < 0.0050 | mg/kg Ts | 30% | EPA 5021 | b) |
| 1,3-Diklorbensen | < 0.0050 | mg/kg Ts | 15% | EPA 5021 | b) |
| 1,3-Diklorpropan | < 0.0050 | mg/kg Ts | 25% | EPA 5021 | b) |
| 1,3-Diklorpropen | < 0.0050 | mg/kg Ts | 25% | EPA 5021 | b) |
| 1,4-Diklorbensen | < 0.0050 | mg/kg Ts | 15% | EPA 5021 | b) |
| 2,2-Diklorpropan | < 0.0050 | mg/kg Ts | 30% | EPA 5021 | b) |
| 2-Klortoluen | < 0.0050 | mg/kg Ts | 30% | EPA 5021 | b) |
| 4-Klortoluen | < 0.0050 | mg/kg Ts | 30% | EPA 5021 | b) |
| Bensen | 0.17 | mg/kg Ts | 25% | EPA 5021 | b) |
| Brombensen | < 0.0050 | mg/kg Ts | 20% | EPA 5021 | b) |
| Bromdiklormetan | < 0.0050 | mg/kg Ts | 25% | EPA 5021 | b) |
| Bromklormetan | < 0.0050 | mg/kg Ts | 30% | EPA 5021 | b) |
| cis-1,2-Dikloreten | < 0.0050 | mg/kg Ts | 30% | EPA 5021 | b) |
| Dibromklormetan | < 0.0050 | mg/kg Ts | 25% | EPA 5021 | b) |
| Dibrommetan | < 0.0050 | mg/kg Ts | 30% | EPA 5021 | b) |
| Diklormetan | 0.016 | mg/kg Ts | 30% | EPA 5021 | b) |
| Etylbensen | 0.0076 | mg/kg Ts | 20% | EPA 5021 | b) |
| Hexaklorbutadien (HCBD) | < 0.0050 | mg/kg Ts | 30% | EPA 5021 | b) |
| iso-Propylbensen | < 0.0050 | mg/kg Ts | 30% | EPA 5021 | b) |
| Klorbensen | < 0.0050 | mg/kg Ts | 25% | EPA 5021 | b) |
| m/p-Xylen | 0.0092 | mg/kg Ts | 30% | EPA 5021 | b) |
| n- Butylbensen | < 0.0050 | mg/kg Ts | 30% | EPA 5021 | b) |
| o- Xylen | < 0.0050 | mg/kg Ts | 30% | EPA 5021 | b) |
| p- Isopropyltoluen | 0.014 | mg/kg Ts | 30% | EPA 5021 | b) |
| Propylbensen | < 0.0050 | mg/kg Ts | 25% | EPA 5021 | b) |
| sec-Butylbensen | < 0.0050 | mg/kg Ts | 30% | EPA 5021 | b) |
| tert-Butylbensen | < 0.0050 | mg/kg Ts | 30% | EPA 5021 | b) |
| Tetrakloreten | < 0.0050 | mg/kg Ts | 20% | EPA 5021 | b) |
| Tetraklormetan | < 0.0050 | mg/kg Ts | 25% | EPA 5021 | b) |
| Toluen | 0.45 | mg/kg Ts | 20% | EPA 5021 | b) |
| trans-1,2-Dikloreten | < 0.0050 | mg/kg Ts | 30% | EPA 5021 | b) |
| trans-1,3-Diklorpropen | < 0.0050 | mg/kg Ts | 25% | EPA 5021 | b) |
| Tribrommetan | < 0.0050 | mg/kg Ts | 30% | EPA 5021 | b) |
| Triklorflourmetan (CFC-11) | < 0.0050 | mg/kg Ts | 30% | EPA 5021 | b) |
| Triklormetan | 0.49 | mg/kg Ts | 25% | EPA 5021 | b) |
| Aluminum Al | 870 | mg/kg Ts | 15% | SS028311 / ICP-AES | b) |
| Arsenik As | < 9.4 | mg/kg Ts | 25% | EN ISO 11885:2009 / SS 028311 utg 1 | b)* |
| Barium Ba | 16 | mg/kg Ts | 25% | EN ISO 11885:2009 / SS 028311 utg 1 | b)* |
| Bly Pb | 5.5 | mg/kg Ts | 25% | EN ISO 11885:2009 / SS 028311 utg 1 | b)* |
| Kadmium Cd | < 0.53 | mg/kg Ts | 25% | EN ISO 11885:2009 / SS 028311 utg 1 | b)* |
| Kobolt Co | < 2.4 | mg/kg Ts | 25% | EN ISO 11885:2009 / SS 028311 utg 1 | b)* |
| Koppar Cu | 24 | mg/kg Ts | 25% | EN ISO 11885:2009 / SS 028311 utg 1 | b)* |
| Krom Cr | 5.2 | mg/kg Ts | 25% | EN ISO 11885:2009 / SS 028311 utg 1 | b)* |
| Kvicksilver Hg | < 0.047 | mg/kg Ts | 20% | SS028311mod/SS-EN ISO17852mod | b)* |
| Nickel Ni | 3 | mg/kg Ts | 25% | EN ISO 11885:2009 / SS 028311 utg 1 | b)* |
| Silver Ag | < 0.94 | mg/kg Ts | 25% | EN ISO 17294-2:2016 / SS 028311, utg 1 | b)* |
| Tenn Sn | 0.97 | mg/kg Ts | 25% | EN ISO 17294-2:2016 / SS 028311, utg 1 | b)* |
| Vanadin V | < 9.4 | mg/kg Ts | 25% | EN ISO 11885:2009 / SS 028311 utg 1 | b)* |
| Zink Zn | 68 | mg/kg Ts | 25% | EN ISO 11885:2009 / SS 028311 utg 1 | b)* |
| Summa Diklorfenoler | < 1.0 | mg/kg Ts | 20% | Internal Method LidMiljö.0A.01.17 | b)* |
| Summa Triklorfenol | < 1.0 | mg/kg Ts | 20% | Internal Method LidMiljö.0A.01.17 | b)* |
| Summa Tetraklorfenol | < 1.0 | mg/kg Ts | 20% | Internal Method LidMiljö.0A.01.17 | b)* |
| Pentaklorfenol | < 1.0 | mg/kg Ts | 20% | Internal Method LidMiljö.0A.01.17 | b)* |
| DDT-o,p | < 0.10 | mg/kg Ts | 20% | Internal Method LidMiljö.0A.01.17 | b)* |
| DDT,p,p'- | < 0.10 | mg/kg Ts | 20% | Internal Method LidMiljö.0A.01.17 | b)* |
| DDE,o,p- | < 0.10 | mg/kg Ts | 20% | Internal Method LidMiljö.0A.01.17 | b)* |
| DDE-p,p | < 0.10 | mg/kg Ts | 20% | Internal Method LidMiljö.0A.01.17 | b)* |
| HCH-alfa | < 0.10 | mg/kg Ts | 20% | Internal Method LidMiljö.0A.01.17 | b)* |
| HCH-beta | < 0.10 | mg/kg Ts | 20% | Internal Method LidMiljö.0A.01.17 | b)* |
| HCH-delta | < 0.10 | mg/kg Ts | 20% | Internal Method LidMiljö.0A.01.17 | b)* |
| HCH,gamma- (Lindane) | < 0.10 | mg/kg Ts | 20% | Internal Method LidMiljö.0A.01.17 | b)* |
| Hexaklorbensen | < 0.10 | mg/kg Ts | 20% | Internal Method LidMiljö.0A.01.17 | b)* |
| Endosulfan-alpha | < 0.10 | mg/kg Ts | 20% | Internal Method LidMiljö.0A.01.17 | b)* |
| Endosulfan-beta | < 0.10 | mg/kg Ts | 20% | Internal Method LidMiljö.0A.01.17 | b)* |
| Endosulfan-sulfate | < 0.10 | mg/kg Ts | 20% | Internal Method LidMiljö.0A.01.17 | b)* |
| Dieldrin | < 0.10 | mg/kg Ts | 20% | Internal Method LidMiljö.0A.01.17 | b)* |
| Endrin | < 0.10 | mg/kg Ts | 20% | Internal Method LidMiljö.0A.01.17 | b)* |
| PCB 28 | < 0.10 | mg/kg Ts | 20% | Internal Method LidMiljö.0A.01.17 | b)* |
| PCB 52 | < 0.10 | mg/kg Ts | 20% | Internal Method LidMiljö.0A.01.17 | b)* |
| PCB 101 | < 0.10 | mg/kg Ts | 20% | Internal Method LidMiljö.0A.01.17 | b)* |
| PCB 118 | < 0.10 | mg/kg Ts | 20% | Internal Method LidMiljö.0A.01.17 | b)* |
| PCB 153 | < 0.10 | mg/kg Ts | 20% | Internal Method LidMiljö.0A.01.17 | b)* |
| PCB 138 | < 0.10 | mg/kg Ts | 20% | Internal Method LidMiljö.0A.01.17 | b)* |
| PCB 180 | < 0.10 | mg/kg Ts | 20% | Internal Method LidMiljö.0A.01.17 | b)* |
| S:a PCB (7st) | < 0.40 | mg/kg Ts |  | Internal Method LidMiljö.0A.01.17 | b)* |
| N-nitroso-di-n-propylamin | < 0.10 | mg/kg Ts | 20% | Internal Method LidMiljö.0A.01.17 | b)* |
| Nitrobensen | < 0.10 | mg/kg Ts | 20% | Internal Method LidMiljö.0A.01.17 | b)* |
| Azobensen | < 0.10 | mg/kg Ts | 20% | Internal Method LidMiljö.0A.01.17 | b)* |
| N-nitrosodifenylamin | < 0.10 | mg/kg Ts | 20% | Internal Method LidMiljö.0A.01.17 | b)* |
| 2,6-Dinitrotoluen | < 0.10 | mg/kg Ts | 20% | Internal Method LidMiljö.0A.01.17 | b)* |
| 2,4-Dinitrotoluen | < 0.10 | mg/kg Ts | 20% | Internal Method LidMiljö.0A.01.17 | b)* |
| Bis(2-kloretyl)eter | < 0.10 | mg/kg Ts | 20% | Internal Method LidMiljö.0A.01.17 | b)* |
| Bis(2-kloroisopropyl)eter | < 0.10 | mg/kg Ts | 20% | Internal Method LidMiljö.0A.01.17 | b)* |
| Hexakloretan | < 0.10 | mg/kg Ts | 20% | Internal Method LidMiljö.0A.01.17 | b)* |
| Isophorone | < 0.10 | mg/kg Ts | 20% | Internal Method LidMiljö.0A.01.17 | b)* |
| Bis(2-kloretoxy)metan | < 0.10 | mg/kg Ts | 20% | Internal Method LidMiljö.0A.01.17 | b)* |
| 2-Klornaftalen | < 0.10 | mg/kg Ts | 20% | Internal Method LidMiljö.0A.01.17 | b)* |
| 4-Klorfenyl fenyleter | < 0.10 | mg/kg Ts | 20% | Internal Method LidMiljö.0A.01.17 | b)* |
| 4-Bromofenyl fenyleter | < 0.10 | mg/kg Ts | 20% | Internal Method LidMiljö.0A.01.17 | b)* |
| Pentaklorbensen | < 0.10 | mg/kg Ts | 20% | Internal Method LidMiljö.0A.01.17 | b)* |
| Dimetylftalat (DMP) | < 0.10 | mg/kg Ts | 20% | Internal Method LidMiljö.0A.01.17 | b)* |
| Dietylftalat | < 0.10 | mg/kg Ts | 20% | Internal Method LidMiljö.0A.01.17 | b)* |
| Di-n-butylftalat | < 0.10 | mg/kg Ts | 20% | Internal Method LidMiljö.0A.01.17 | b)* |
| Bensylbutylftalat | < 0.10 | mg/kg Ts | 20% | Internal Method LidMiljö.0A.01.17 | b)* |
| Di-(2-etylhexyl)ftalat | < 1.0 | mg/kg Ts | 20% | Internal Method LidMiljö.0A.01.17 | b)* |
| Di-n-oktylftalat | < 0.10 | mg/kg Ts | 20% | Internal Method LidMiljö.0A.01.17 | b)* |
| Metylkvicksilver | 3.1 | µg/kg Ts |  | EPA Method 1630 | c)* |
| 1-(3,4-Dichlorophenyl)-3-methylurea | <1.0 | µg/kg Ts | 27% | J. of Chromatogr. A, 1217 (2010) 2933–2939 mod. | a) |
| 1-(3,4-Dichlorophenyl)urea | <1.0 | µg/kg Ts | 27% | J. of Chromatogr. A, 1217 (2010) 2933–2939 mod. | a) |
| 2,4,5-T | <10 | µg/kg Ts | 27% | J. of Chromatogr. A, 1217 (2010) 2933–2939 mod. | a) |
| 2,4-D | <10 | µg/kg Ts | 27% | J. of Chromatogr. A, 1217 (2010) 2933–2939 mod. | a) |
| 2,4-Dichlorprop | <10 | µg/kg Ts | 27% | J. of Chromatogr. A, 1217 (2010) 2933–2939 mod. | a) |
| 2,6-Dichlorobenzamide | <10 | µg/kg Ts | 27% | J. of Chromatogr. A, 1217 (2010) 2933–2939 mod. | a) |
| Atrazine | <10 | µg/kg Ts | 27% | J. of Chromatogr. A, 1217 (2010) 2933–2939 mod. | a) |
| Atrazine-desethyl | <10 | µg/kg Ts | 27% | J. of Chromatogr. A, 1217 (2010) 2933–2939 mod. | a) |
| Atrazine-desisopropyl | <10 | µg/kg Ts | 27% | J. of Chromatogr. A, 1217 (2010) 2933–2939 mod. | a) |
| Bentazone | <10 | µg/kg Ts | 27% | J. of Chromatogr. A, 1217 (2010) 2933–2939 mod. | a) |
| Cyanazine | <10 | µg/kg Ts | 27% | J. of Chromatogr. A, 1217 (2010) 2933–2939 mod. | a) |
| Diuron | <1.0 | µg/kg Ts | 27% | J. of Chromatogr. A, 1217 (2010) 2933–2939 mod. | a) |
| Imazapyr | <10 | µg/kg Ts | 27% | J. of Chromatogr. A, 1217 (2010) 2933–2939 mod. | a) |
| Linuron | <10 | µg/kg Ts | 27% | J. of Chromatogr. A, 1217 (2010) 2933–2939 mod. | a) |
| MCPA | <10 | µg/kg Ts | 27% | J. of Chromatogr. A, 1217 (2010) 2933–2939 mod. | a) |
| Mecoprop | <10 | µg/kg Ts | 27% | J. of Chromatogr. A, 1217 (2010) 2933–2939 mod. | a) |
| Simazine | <10 | µg/kg Ts | 27% | J. of Chromatogr. A, 1217 (2010) 2933–2939 mod. | a) |
| Terbuthylazine | <10 | µg/kg Ts | 27% | J. of Chromatogr. A, 1217 (2010) 2933–2939 mod. | a) |

Non-accredited analyzes are marked with *

The measurement uncertainty, unless otherwise stated, is reported as extended measurement uncertainty with a coverage factor of 2. Increased reporting limit for PAHs, aliphatics and aromatics due to difficult sample matrix.

1. Eurofins Food & Feed Testing Sweden (Lidköping), SWEDEN, ISO/IEC 17025:2017 SWEDAC 1977
2. Eurofins Environment Testing Sweden AB, SWEDEN, ISO/IEC 17025:2017 SWEDAC 1125
3. IVL Svenska Miljöinstitutet AB, SWEDEN

**Table SM-3.** PAHs, and aliphatic hydrocarbons results after treatment, and fiberbanks as initial concentrations before treatment.

| **Species** | ***B adusta*** | ***C. muraii*** | ***D. crustulinus*** | ***G. applanatum*** | ***H. annosum*** | ***H. tabacina*** | ***L. sulphureus*** | ***P. ferrugineofuscus*** | ***P. punctatus*** | ***P. tremellosa*** | ***P. gigantea*** | ***S. odora*** | ***S. sanguinolentum*** | ***T. hirsuta*** | ***T. ochracea*** | ***Fiberbank autoclaved*** |
| --- | --- | --- | --- | --- | --- | --- | --- | --- | --- | --- | --- | --- | --- | --- | --- | --- |
| Naphthalene (mg/kg) | 15.231 | 12.616 | 11.64 | 14.542 | 20.468 | 15.903 | 16.364 | 18.169 | 5.271 | 21.837 | 18.508 | 20.123 | 18.367 | 20.97 | 18.572 | 23.511 |
| Acenaphthylene (mg/kg) | 0.759 | 0.819 | 0.621 | 0.765 | 0.825 | 1.089 | 1.091 | 1.041 | 0.566 | 0.953 | 0.793 | 1.069 | 0.81 | 1.232 | 0.835 | 1.104 |
| Acenaphthene (mg/kg) | 0.762 | 1.16 | 0.895 | 1.171 | 0.896 | 1.694 | 1.627 | 1.522 | 0.76 | 1.06 | 0.88 | 1.681 | 0.831 | 1.754 | 0.893 | 1.739 |
| Fluorene (mg/kg) | 0.377 | 0.749 | 0.563 | 0.727 | 0.455 | 1.019 | 0.911 | 0.95 | 0.483 | 0.532 | 0.459 | 1.045 | 0.425 | 0.986 | 0.463 | 1.014 |
| Phenanthrene (mg/kg) | 1.595 | 1.772 | 1.5 | 1.807 | 1.757 | 2.72 | 2.536 | 2.151 | 1.402 | 1.987 | 1.694 | 2.657 | 1.737 | 2.959 | 1.761 | 2.588 |
| Anthracene (mg/kg) | 0.266 | 0.314 | 0.198 | 0.302 | 0.283 | 0.524 | 0.511 | 0.374 | 0.267 | 0.333 | 0.257 | 0.535 | 0.278 | 0.604 | 0.266 | 0.493 |
| Fluoranthene (mg/kg) | 0.417 | 0.379 | 0.3 | 0.387 | 0.905 | 0.733 | 0.686 | 0.511 | 0.356 | 0.415 | 0.473 | 0.892 | 0.457 | 0.78 | 0.524 | 0.586 |
| Pyrene (mg/kg) | 0.519 | 0.433 | 0.358 | 0.476 | 1.054 | 0.915 | 0.762 | 0.591 | 0.438 | 0.372 | 0.56 | 1.446 | 0.606 | 0.95 | 0.721 | 0.563 |
| Benz[a]anthracene (mg/kg) | < 0,002 | 0.059 | 0.053 | 0.058 | 0.153 | 0.087 | 0.097 | 0.064 | 0.058 | < 0,002 | < 0,002 | 0.137 | < 0,002 | 0.108 | < 0,002 | 0.092 |
| Chrysene (mg/kg) | 0.135 | 0.094 | 0.093 | 0.124 | 0.26 | 0.18 | 0.221 | 0.097 | 0.084 | < 0,003 | < 0,003 | 0.21 | < 0,003 | 0.19 | < 0,003 | 0.197 |
| Benzo[o]fluoranthene (mg/kg) | < 0,006 | < 0,006 | < 0,006 | < 0,006 | < 0,006 | < 0,006 | 0.085 | 0.068 | < 0,006 | < 0,006 | < 0,006 | < 0,006 | < 0,006 | < 0,006 | < 0,006 | < 0,006 |
| Benzo[k]fluoranthene (mg/kg) | < 0,004 | 0.043 | < 0,004 | < 0,004 | < 0,004 | < 0,004 | < 0,004 | 0.059 | < 0,004 | < 0,004 | < 0,004 | < 0,004 | < 0,004 | < 0,004 | < 0,004 | < 0,004 |
| Benz(a)pyrene (mg/kg) | < 0,049 | < 0,049 | < 0,049 | < 0,049 | < 0,049 | < 0,049 | < 0,049 | < 0,049 | < 0,049 | < 0,049 | < 0,049 | < 0,049 | < 0,049 | < 0,049 | < 0,049 | < 0,049 |
| Indeno[1,2,3-cd]pyrene (mg/kg) | < 0,002 | 0.016 | < 0,002 | < 0,002 | < 0,002 | < 0,002 | < 0,002 | 0.012 | < 0,002 | < 0,002 | < 0,002 | < 0,002 | < 0,002 | < 0,002 | < 0,002 | < 0,002 |
| Dibenz[a,h]anthracene (mg/kg) | < 0,002 | 0.009 | < 0,002 | < 0,002 | < 0,002 | 0.034 | < 0,002 | 0.021 | < 0,002 | < 0,002 | < 0,002 | < 0,002 | < 0,002 | < 0,002 | < 0,002 | < 0,002 |
| Benzo[ghi]perylene (mg/kg) | < 0,001 | 0.017 | < 0,001 | < 0,001 | < 0,001 | < 0,001 | < 0,001 | < 0,001 | < 0,001 | < 0,001 | < 0,001 | < 0,001 | < 0,001 | < 0,001 | < 0,001 | < 0,001 |
| Decane (mg/kg) | < 0,073 | 0.713 | 0.99 | 1.307 | < 0,073 | < 0,073 | < 0,073 | 0.574 | 0.517 | < 0,073 | < 0,073 | < 0,073 | < 0,073 | < 0,073 | < 0,073 | < 0,073 |
| Undecane (mg/kg) | < 0,25 | 0.914 | 1.31 | 1.282 | < 0,25 | < 0,25 | < 0,25 | 1.091 | < 0,25 | < 0,25 | < 0,25 | < 0,25 | < 0,25 | < 0,25 | < 0,25 | < 0,25 |
| Dodecane (mg/kg) | < 0,33 | 3.942 | 5.308 | 4.988 | < 0,33 | < 0,33 | < 0,33 | 4.525 | 2.612 | < 0,33 | < 0,33 | < 0,33 | < 0,33 | < 0,33 | < 0,33 | < 0,33 |
| Tridecane (mg/kg) | < 0,295 | 2.488 | 2.202 | 2.339 | < 0,295 | < 0,295 | < 0,295 | 3.151 | < 0,295 | < 0,295 | < 0,295 | < 0,295 | < 0,295 | < 0,295 | < 0,295 | < 0,295 |
| Tetradecane (mg/kg) | < 0,656 | 3.748 | 4.548 | 4.016 | < 0,656 | < 0,656 | < 0,656 | 2.903 | 0.974 | < 0,656 | < 0,656 | < 0,656 | < 0,656 | < 0,656 | < 0,656 | < 0,656 |
| Pentadecane (mg/kg) | < 0,192 | 1.282 | 0.95 | 0.261 | < 0,192 | < 0,192 | < 0,192 | 1.03 | < 0,192 | < 0,192 | < 0,192 | < 0,192 | < 0,192 | < 0,192 | < 0,192 | < 0,192 |
| Hexadecane (mg/kg) | < 0,963 | < 0,963 | < 0,963 | < 0,963 | < 0,963 | < 0,963 | < 0,963 | 2.172 | < 0,963 | < 0,963 | < 0,963 | < 0,963 | < 0,963 | < 0,963 | < 0,963 | < 0,963 |
| Octadecane (mg/kg) | < 1,099 | < 1,099 | < 1,099 | < 1,099 | < 1,099 | < 1,099 | < 1,099 | < 1,099 | < 1,099 | < 1,099 | < 1,099 | < 1,099 | < 1,099 | < 1,099 | < 1,099 | < 1,099 |
| Nonadecane (mg/kg) | < 0,122 | 0.529 | < 0,122 | < 0,122 | < 0,122 | < 0,122 | < 0,122 | 0.897 | < 0,122 | < 0,122 | < 0,122 | < 0,122 | < 0,122 | < 0,122 | < 0,122 | < 0,122 |
| Eicosane (mg/kg) | < 0,771 | 1.234 | < 0,771 | < 0,771 | < 0,771 | < 0,771 | < 0,771 | 1.672 | < 0,771 | < 0,771 | < 0,771 | < 0,771 | < 0,771 | < 0,771 | < 0,771 | < 0,771 |
| Heneicosane (mg/kg) | < 0,122 | < 0,122 | 0.935 | 0.836 | < 0,122 | < 0,122 | < 0,122 | 19.391 | < 0,122 | < 0,122 | < 0,122 | < 0,122 | < 0,122 | < 0,122 | < 0,122 | < 0,122 |
| Docosane (mg/kg) | < 0,572 | < 0,572 | < 0,572 | < 0,572 | < 0,572 | < 0,572 | < 0,572 | 2.568 | < 0,572 | < 0,572 | < 0,572 | < 0,572 | < 0,572 | < 0,572 | < 0,572 | < 0,572 |
| Tricosane (mg/kg) | < 0,287 | 1.974 | 1.55 | 1.336 | < 0,287 | < 0,287 | < 0,287 | 1.29 | < 0,287 | < 0,287 | < 0,287 | < 0,287 | < 0,287 | < 0,287 | < 0,287 | < 0,287 |
| Tetracosane (mg/kg) | < 0,58 | 0.739 | < 0,58 | < 0,58 | < 0,58 | < 0,58 | < 0,58 | < 0,58 | < 0,58 | < 0,58 | < 0,58 | < 0,58 | < 0,58 | < 0,58 | < 0,58 | < 0,58 |

The results for all control samples, which utilized perlite, were measured to be below the quantification limit for all analytes. Therefore, they were not included in the corresponding table.

**Table SM-4.**p-Values from paired samples Student´s t-test. p-Values obtained from the paired samples Student's t-test are used to determine statistical significance, with values below 0.05 considered significant and those above 0.05 deemed not significant.

| **Species** | PAHs 2-3 rings | PAHs 4-6 rings | Aliphatics (C 10-16)* | Aliphatics (C 18-24)* |  |
| --- | --- | --- | --- | --- | --- |
| *B. adusta* | 0.08538 | 0.0733 | NA | NA | |
| *C. muraii* | 0.02884 | 0.2461 | 0.08701 | NA | |
| *D. crustulinus* | 0.006906 | 0.01957 | 0.00355 | NA | |
| *G. applanatum* | 0.001608 | 0.1173 | 0.0012 | NA | |
| *H. annosum* | 0.01295 | 0.0226 | NA | NA | |
| *H. tabacina* | 0.8073 | 0.2137 | NA | NA | |
| *L. sulphureus* | 0.08121 | 0.1067 | NA | NA | |
| *P. ferrugineofuscus* | 0.2912 | 0.4877 | 0.3257 | NA | |
| *P. punctatus* | 0.01161 | 0.1111 | 0.198 | NA | |
| *P. tremellosa* | 0.01536 | 0.007459 | NA | NA | |
| *P. gigantea* | 0.02688 | 0.022 | NA | NA | |
| *S. odora* | 0.4871 | 0.04053 | NA | NA | |
| *S. sanguinolentum* | 0.02581 | 0.1395 | NA | NA | |
| *T. hirsuta* | 0.05185 | 0.009922 | NA | NA | |
| *T. ochracea* | 0.1062 | 0.06435 | NA | NA | |
